# Índice de Triglicerídeos-Glicose e Fluxo Lento Coronariano: Uma Nova Ferramenta Diagnóstica?

**DOI:** 10.36660/abc.20230373

**Published:** 2023-07-19

**Authors:** Maria Cristina Costa de Almeida, Marildes Luiza de Castro

**Affiliations:** 1 Centro Universitário de Belo Horizonte Belo Horizonte MG Brasil Centro Universitário de Belo Horizonte, Belo Horizonte, MG – Brasil; 2 Hospital de Clínicas Universidade Federal de Minas Gerais Belo Horizonte MG Brasil Hospital de Clínicas – Universidade Federal de Minas Gerais (UFMG), Belo Horizonte, MG – Brasil

**Keywords:** Índice de Triglicerideos-glucose, Fluxo Lento Coronariano, Resistência Insulinica, Doença Cardiovascular

A doença cardiovascular (DCV) é a principal causa de morte e incapacidade em todo o mundo, sendo responsável pela maioria das mortes por doenças não transmissíveis.^1^ Fatores de risco importantes como tabagismo, diabetes mellitus (DM), hipertensão ou dislipidemia chamam mais a atenção dos médicos. Entretanto, indivíduos podem desenvolver aterosclerose subclínica mesmo que não apresentem sintomas ou fatores de risco cardiovasculares tradicionais, pois é uma doença lentamente progressiva, com artérias acometidas por espessamento, rigidez, perda de elasticidade e aumento da fragilidade da parede à estenose e oclusão do lúmen.^2^

A resistência à insulina (RI) pode ser um dos mecanismos potenciais do desenvolvimento de DCV, sendo reconhecida como um indicador de inflamação sistêmica e distúrbios metabólicos intimamente relacionados à DCV aterosclerótica. Além disso, é considerada um fator de alto risco para DM e DCV.^1^

Os métodos atuais para avaliar a RI incluem teste de clamp euglicêmico hiperinsulinêmico (CEH) e a RI estimada pelo modelo de avaliação da homeostase (HOMA-IR), mas seu uso clínico é limitado devido ao tempo e ao custo.

Espera-se que um novo método, o índice de triglicerídeos-glicose (TyGI), se torne um índice alternativo para medir a RI.^1^ É um parâmetro sintético da glicemia de jejum em indivíduos saudáveis, considerado um marcador substituto confiável de RI e intimamente relacionado com DCV, assim como a RI pode prejudicar a função endotelial coronariana por meio do estresse oxidativo e indução de inflamação.^1^

O uso de TyGI parece simplificar o diagnóstico de intolerância à glicose. Mais e mais estudos descobriram que o TyGI não está apenas significativamente associado ao risco de aterosclerose, DM e doença arterial coronariana, mas seus níveis elevados aumentam o mau prognóstico de doenças cardiovasculares, como reestenose intra-stent e fibrilação atrial.^3^

A investigação da relação entre TyGİ e fluxo lento coronariano (FLC) pode ser de grande interesse na prática clínica, pois estudos têm demonstrado forte associação entre RI e FLC, considerando a RI como fator de risco independente para FLC em pacientes com diagnóstico de intolerância à glicose.^4 , 5^

A rigidez arterial é um dos primeiros tipos de dano funcional que ocorre durante o processo de envelhecimento vascular, durante o qual a elasticidade arterial diminui.^6^ Evidências crescentes sugerem que a rigidez arterial é um poderoso preditor de risco futuro de doenças cardiovasculares, como síndromes coronarianas agudas, insuficiência cardíaca e AVC isquêmico ou hemorrágico.^6^

Como a comunidade médica sabe, o FLC é a opacificação tardia da vasculatura coronária no nível distal e pode se apresentar como síndromes coronarianas agudas e morte súbita cardíaca. Diferentes hipóteses têm sido postuladas sobre seu mecanismo, como disfunção microvascular e endotelial, com aumento do tônus vasomotor de repouso e tendência ao vasoespasmo. Sua incidência é de cerca de 1-5% dos pacientes submetidos à cineangiocoronariografia e tem sido mais frequentemente encontrada em homens jovens tabagistas com síndrome metabólica.^7^

Além disso, o TyGI é considerado um marcador de aterosclerose subclínica, relacionado ao grau de calcificação da artéria coronária e à espessura médio-intimal da carótida. Além disso, há correlação positiva com pior prognóstico em pacientes com infarto agudo do miocárdio quando seu nível ultrapassa 9,75, o que pode servir de limiar para avaliação de isquemia coronariana residual.^3^

O nível de TyGI acima de 9,75 aumentou o risco de razão de fluxo quantitativo pós-intervenção coronariana percutânea ≤ 0,92, o que tem um prognóstico ruim. Portanto, TyGI acima de 9,75 pode ser usado como um limiar para terapia medicamentosa intensiva para melhorar a isquemia coronariana após intervenção coronariana percutânea.

O TyGI é um índice sanguíneo fácil, reprodutível, prático e convenientemente mensurável que pode avaliar o perfil cardiometabólico e a função fisiológica coronária.
