# Supplementary material for: Índice de Triglicerídeos-Glicose e Fluxo Lento Coronariano: Uma Nova Ferramenta Diagnóstica?
Source: Arq Bras Cardiol. 2023 Jul 19;120(6):e20230373. [Article in Portuguese] doi: 10.36660/abc.20230373 (PMC10389683; doi:10.36660/abc.20230373)
Supplement: Translation

# Translation: Triglycerides-Glucose İndex and Coronary Slow Flow: A New Diagnostic Tool?

**Authors:** Maria Cristina Costa de Almeida, Marildes Luiza de Castro

**Affiliations:** 1 Brazil Centro Universitário de Belo Horizonte, Belo Horizonte, MG – Brazil; 2 Brazil Hospital de Clínicas – Universidade Federal de Minas Gerais (UFMG), Belo Horizonte, MG – Brazil

**Keywords:** Triglyceride-glucose Index, Coronary Slow Flow, Insulin Resistance, Cardiovascular Disease

Cardiovascular disease (CVD) is the primary cause of death and disability worldwide, accounting for the majority of deaths from noncommunicable diseases.^1^ Important risk factors such as smoking, diabetes mellitus (DM), hypertension, or dyslipidemia draw more attention from physicians. However, individuals can develop subclinical atherosclerosis even if they do not have traditional cardiovascular risk factors or symptoms since it is a slowly progressive disease, with arteries affected by thickening, stiffness, loss of elasticity, and increased wall fragility to stenosis and lumen occlusion.^2^

Insulin resistance (IR) may be one of the potential mechanisms of the development of CVD, being recognized as an indicator of systemic inflammation and metabolic disorders closely related to atherosclerotic CVD. Moreover, it is considered a high-risk factor for DM and CVD.^1^

The current methods for assessing IR include the hyperinsulinemia-euglycemic (HIEG) clamp test and homeostasis model assessment-estimated IR (HOMA-IR), but their clinical use is limited due to time-consuming and expensive.

A novel method, the triglyceride-glucose index (TyGI), is expected to become an alternative index for measuring IR.^1^ It is a synthetic parameter of fasting glucose in healthy individuals, considered a reliable surrogate marker of IR, and closely related to CVD, as IR can damage coronary endothelial function through oxidative stress and inducing inflammation.^1^

Using TyGI seems to simplify the diagnosis of impaired glucose tolerance (IGT). More and more studies have found that the TyGI is not only significantly associated with the risk of atherosclerosis, DM, and coronary artery disease, but its elevated levels increase the poor prognosis of CVDs such as in-stent restenosis and atrial fibrillation.^3^

The investigation of the relationship between TyGI and coronary slow flow (CSF) could be of great interest in clinical practice, as studies have demonstrated a strong association between IR and CSF, considering IR as an independent risk factor for CSF in patients with IGT.^4 , 5^

Arterial stiffness is one of the earliest types of functional damage that occurs during the vascular aging process, during which arterial elasticity decreases.^6^ Growing evidence suggests that arterial stiffness is a powerful predictor for future risk of CVDs such as acute coronary syndromes, heart failure, and ischemic or hemorrhagic stroke.^6^

As the medical community knows, CSF is the delayed opacification of the coronary vasculature at the distal level and can present as acute coronary syndromes and sudden cardiac death. Different hypotheses have been postulated about its mechanism, such as microvascular and endothelial dysfunction, with increased resting vasomotor tone and a tendency to vasospasm. Its incidence is about 1-5% of patients submitted to coronary angiography and has been most frequently found in young men who are smokers with metabolic syndrome.^7^

Moreover, TyGI is considered a marker of subclinical atherosclerosis related to the degree of coronary artery calcification and carotid intima-media thickness. Furthermore, there is a positive correlation with poor prognosis in patients with acute myocardial infarction when its level exceeds 9.75, which may serve as a threshold for assessing residual coronary ischemia.^3^

The TyGI level above 9.75 increased the risk of post-percutaneous coronary intervention quantitative flow ratio ≤ 0.92, which has a poor prognosis. Therefore, TyGI above 9.75 may be used as a threshold for intensive drug therapy to improve coronary ischemia after percutaneous coronary intervention.

The TyGI is an easy, reproducible, practical, and conveniently measurable blood index that can assess cardiometabolic profile and coronary physiological function.
